# Epidemiological Trend of Typhoid and Paratyphoid Fevers in Zhejiang Province, China from 1953 to 2014

**DOI:** 10.3390/ijerph15112427

**Published:** 2018-11-01

**Authors:** Hua Gu, Congcong Yan, Zhenggang Jiang, Xiuyang Li, Enfu Chen, Jianmin Jiang, Qingwu Jiang, Yibiao Zhou

**Affiliations:** 1 Zhejiang Provincial Center for Disease Control and Prevention, Hangzhou 310051, China; kjzxhgu@163.com (H.G.); zhgjiang@cdc.zj.cn (Z.J.); enfchen@cdc.zj.cn (E.C.); jmjiang@cdc.zj.cn (J.J.); 2 Zhejiang Provincial Center for Medical Science Technology & Education, Hangzhou 310006, China; 3 Department of Preventive Medicine, School of Medicine, Ningbo University, Ningbo 315211, China; kb981147121@163.com; 4 Department of Epidemiology & Health Statistics, Zhejiang University, Hangzhou 310058, China; lixiuyang@zju.edu.cn; 5 Department of Epidemiology & Health Statistics, Fudan University, Shanghai 200032, China; jiangqw@fudan.edu.cn

**Keywords:** typhoid, paratyphoid, epidemiological characteristics

## Abstract

*Background*: The incidences of typhoid and paratyphoid remain high and these diseases still pose a public health problem in China and in Zhejiang Province in particular. This study aimed to investigate the trend of typhoid and paratyphoid in Zhejiang Province from 1953 to 2014 and to provide a theoretical basis for the prevention and control of these diseases. *Methods*: Included in this study were compiled epidemiological data of typhoid and paratyphoid cases in Zhejiang from 1953 to 2003 and epidemiological data of those from 2004 to 2014 registered in the China Information System for Diseases Control and Prevention. Description methods were employed to explore the epidemiological characteristics, including long-term trend, gender distribution, age distribution, and occupation distribution. Incidence maps were made to represent the annual average incidences for each municipality. Spearman’s rank correlation was performed to detect the correlation between incidence and average elevation, and circular distribution was calculated to identify the seasonality and peak days of the diseases. A *p*-value of <0.05 was considered statistically significant. *Results*: A total of 182,602 typhoid and paratyphoid cases were reported in Zhejiang Province from 1953 to 2014, and the average annual incidence was 7.89 per 100,000 population. The incidence in 2014 decreased by 93.82% compared with that in 1953 and by 95.00% compared with the highest incidence rate. The average incidence before 2003 was negatively correlated with the average elevation of each region in Zhejiang province (*r* < 0, *p* < 0.05), but there was no statistically significant correlation from 2003. The peak period of diseases fell in the months from April to October every year. The incidence among the population group aged over 35 rose gradually but declined sharply among those between 20 and 34. *Conclusions*: The incidence of typhoid and paratyphoid decreased in Zhejiang Province from 1953 to 2014 but remained high in some regions. Proper measures for prevention and control are warranted in the southeast coast areas and for high-risk populations.

## 1. Introduction

Typhoid and paratyphoid are intestinal infections caused by the bacteria *Salmonella* Typhi and Paratyphi and are transmitted by the fecal–oral route. Their main symptoms are digestive tract reaction. Infection may occur after consumption of food or water contaminated by feces or urine [1]. The incubation period is mostly 3–42 days with an average of 14 days for typhoid fever and 2–15 days for paratyphoid. Their clinical manifestations are varied, including fever, fatigue, headaches, gastrointestinal reaction, and complications may develop in severe cases, such as intestinal bleeding and perforation [1]. The World Health Organization (WHO) estimated that, globally, typhoid and paratyphoid patients in 2000 totaled 21.65 million, of whom 220,000 died [2], and the number declined to 13.43 million in 2010 [3]. The incidences of typhoid and paratyphoid in developed countries declined steadily by 0.13–1.2 per 100,000 as a result of a series of measures taken for water quality control and improved health facilities [4]. Conditions favorable for these diseases are still common in Asia, Africa, and poor countries and thus have a great impact on social and economic development. China has always been among the high-incidence areas, with an incidence rate of about 10–50/100,000 before 1990, which dropped to 1.28/100,000 in 2009 [5,6]. Zhejiang Province is economically developed, but historical incidences were higher there than the national average [5,7,8]. Although the incidence rate in this province has fallen, typhoid and paratyphoid continue to be problematic intestinal diseases.

The geographical information system (GIS) is a widely used tool to explore the spatiotemporal characteristics of infectious diseases, which could help to monitor and prevent communicable diseases [9]. Also, it makes it easy for researchers to identify the incidence difference of any infectious disease between regions via an incidence level map [10,11,12,13].

In this paper, we aimed to investigate the epidemiological characteristics of typhoid and paratyphoid fevers and to detect high-risk populations and areas, thus providing a basis for the prevention and monitoring of typhoid and paratyphoid in Zhejiang Province.

## 2. Methods

### 2.1. Data Sources

Retrieved for this study was a compilation of epidemiological data in Zhejiang Province from 1953 to 2003, the data from the China Information System for Diseases Control and Prevention since 1 January 2004, and demographic data from the Statistical Yearbooks of Zhejiang Province. Detailed information, such as age, gender, occupation, and some other information of patients with typhoid and paratyphoid, had not been classified until 1991. According to other studies, the incidences of typhoid and paratyphoid in the southwest areas were lower than those in other places. Therefore, we explored the correlation between their incidences and altitude.

The cases included in this study were individuals registered in the China Information System for Diseases Control and Prevention and diagnosed as typhoid or paratyphoid using the unified diagnostic criteria in Zhejiang Province from 1953 to 2014. All patients were diagnosed using criteria promulgated by the Ministry of Health of the People’s Republic of China [14]. Details are as follows: the patient had fever of unknown origin, accompanied by a fourfold or greater increase of specific antibody titer in the recovery serum as against the acute phase, or the patient had unexplained fever, and *S.* Typhi or *S.* Paratyphi could be isolated in any specimen of serum, bone marrow, feces, or bile [14].

### 2.2. Study Area

**Figure 1:**
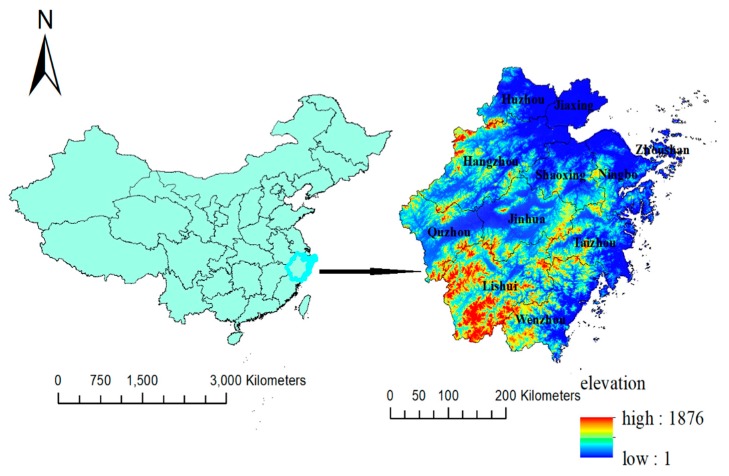
Location and elevation of Zhejiang Province at the municipal level. (Blue represents the low elevation, and the darker the color, the lower the elevation. Red represents high elevation, and the darker the color, the higher the elevation).

Zhejiang (27°12′–31°31′ North, 118°–123° East) is located on the southeastern coast of China and has a subtropical monsoon climate with abundant rainfall. It governed nine provincial municipalities before 1983, with Huzhou being detached from Jiaxing in 1983 and Quzhou from Jinhua in 1985. Thus, now it governs 11 provincial municipalities (Figure 1).

### 2.3. Statistical Software

SPSS (version 16.0, IBM Inc., Chicago, IL, USA) was employed for Spearman’s rank correlation. All results were considered statistically significant if *p* < 0.05 for both sides. ArcGIS software (version 10.1, ESRI Inc.; Redlands, CA, USA) was used to for mapping incidences.

#### Circular Distribution

Circular distribution was calculated for the monthly incidence and the peak day. The seasonality of disease within a year could be explained by the *r*-value. Circular distribution is generally applicable to a seasonal disease with only one peak period [15]. To calculate circular distribution, 365 days in a year is changed to 360°, with one day being represented by 0.9863°, and the monthly median is taken as the middle value of the group and then converted into degrees [16], hence, the following formulas:

(1) $$r=\sqrt{{\left[\left(\sum f_{i}\cos\alpha_{i}\right)/n\right]}^{2}+{\left[\left(\sum f_{i}\sin\alpha_{i}\right)/n\right]}^{2}}$$

(2) $$s=\frac{180^{{}^{\circ}}}{\pi }\sqrt{-2\ln r}$$

where *f_i_* is the monthly cases of disease, *α_i_* is the monthly degree, *r*-value is the index for degree of dispersion, and *s* is the standard deviation of the angle.

The data generated during the study are not publicly available due to regulations, but they can be obtained from the corresponding author on request.

## 3. Results

### 3.1. Epidemiological Characteristics from 1953 to 2014

#### 3.1.1. Overall Epidemiological Trend

A total of 182,602 typhoid and paratyphoid cases were reported in Zhejiang Province in 62 years from 1953 to 2014, with annual incidences ranging from 0.95 per 100,000 to 19.04 per 100,000 and the average annual incidence being 7.89 per 100,000 (adding the annual incidence rate to the average). The incidence of typhoid and paratyphoid in 2014 decreased by 14.45 per 100,000 as against that in 1953 and by 18.09 per 100,000 as against the highest. 

**Figure 2:**
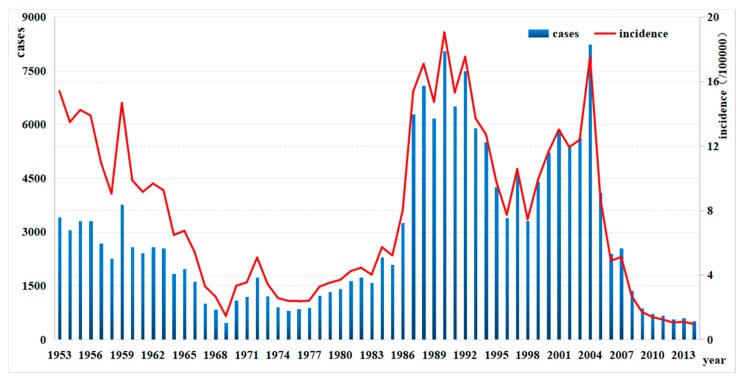
Cases and incidence trend of typhoid and paratyphoid in Zhejiang from 1953 to 2014.

Several large fluctuations could be seen in the incidence of typhoid and paratyphoid in the province from 1953 to 2014: (1) a slowly downward trend from 1953 to 1983 with a sudden rise in 1959; (2) a rising/falling pattern from 1984 to 2004 with a peak in 1990 and another in 2004; and (3) a sharp falling after 2004 to 0.95 per 100,000 population in 2014 (Figure 2).

#### 3.1.2. Altitude Distribution

**Figure 3:**
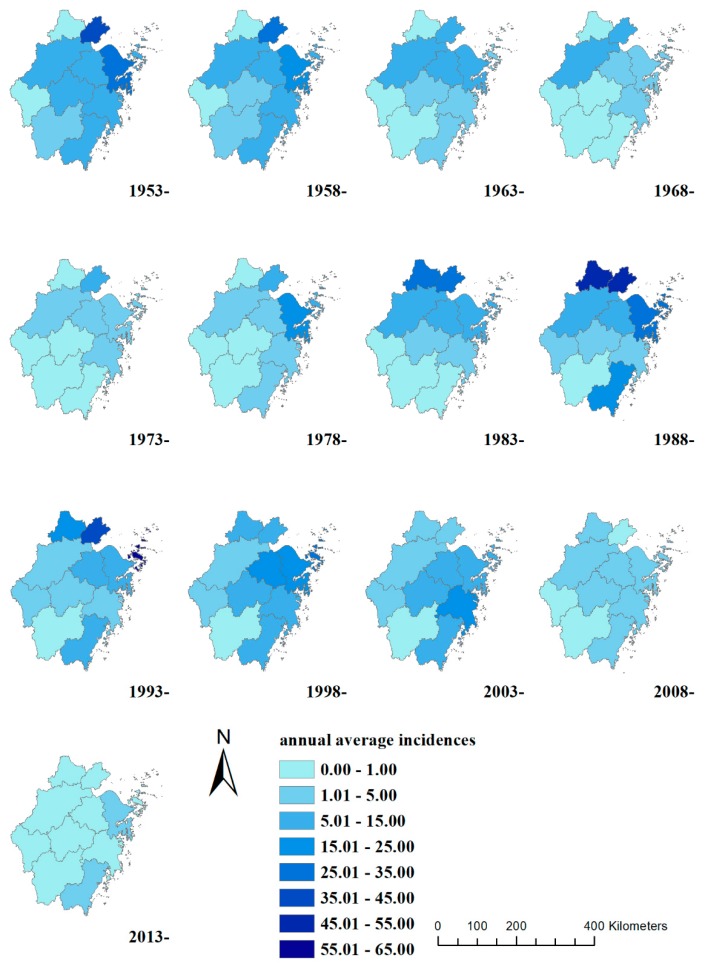
Annual average incidences of typhoid and paratyphoid in Zhejiang Province from 1953 to 2014. (Huzhou being detached from Jiaxing in 1983, and the incidences in Huzhou being 0.00 per 100,000 population before 1983; Quzhou being detached from Jinhua in 1985, and the incidences in Huzhou being 0.00 per 100,000 population before 1985).

The incidence of typhoid and paratyphoid was lower at high altitudes as in Lishui, Quzhou, and Jinhua but a little bit higher at low altitudes in the north areas like Jiaxing, Huzhou, and Shaoxing (Figure 1 and Figure 3). Great changes could be observed from 2000 to 2014. The incidence in the southeast coastal areas such as Ningbo, Zhoushan, Taizhou, and Wenzhou was much higher than the other areas, declined obviously in the north areas, and remained always low at high altitudes in the southwest province. From 1953 to 2002, the average incidence of typhoid and paratyphoid was negatively correlated with the average elevation of each region in Zhejiang Province, and the Spearman’s rank correlation was statistically significant, but there was no statistically significant correlation in 2003 between the incidence and elevation (Table 1).

#### 3.1.3. Regional Distribution

The incidence of typhoid and paratyphoid has generally declined since 1953 (Table 2). The average annual incidence showed a downward trend from 1953 to 1977, with the highest being found in Ningbo, Jiaxing, and Taizhou. The annual incidence greatly rose in all municipalities expect Jinhua and Lishui from 1978 to 1987, was higher than 50.00 per 100,000 in Jiaxing and Huzhou from 1988 to 1992, and rose to 63.92 per 100,000 in Zhoushan from 1993 to 1997.

**Figure 4:**
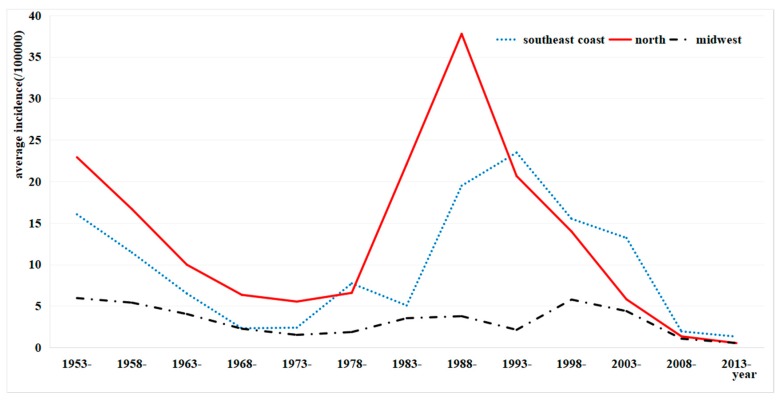
The average annual incidence of typhoid and paratyphoid in three areas in Zhejiang from 1953 to 2014.

The incidence in Ningbo, Huzhou, Jiaxing, Shaoxing, and Zhoushan had an obvious falling/rising pattern and the second peak was higher than the first. The incidence in Lishui and Quzhou was always at a low level (Figure 3). The incidence of typhoid and paratyphoid showed a rising/falling pattern in almost all municipalities. The average annual incidence in the north areas of the province was higher than the southeast, and that in the southeast coastal province higher than the mid-west before 1993, but the incidence in the southeast coastal areas became higher than that in the north and mid-west in 1993 (Figure 4).

### 3.2. Epidemiological Characteristics from 1991 to 2014

#### 3.2.1. Temporal Distribution

**Figure 5:**
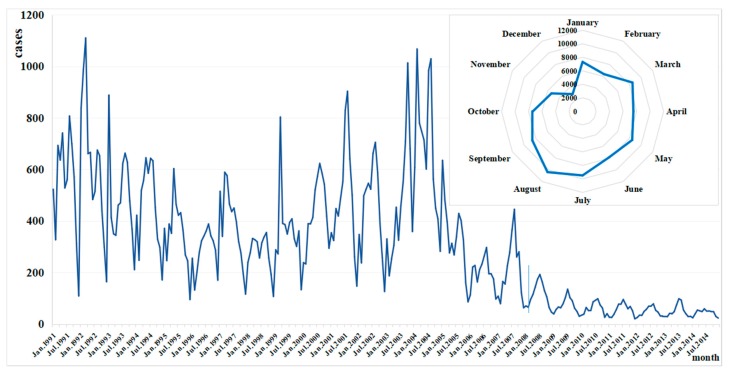
Monthly distribution of typhoid and paratyphoid cases in Zhejiang from 1991 to 2014.

The cases began to increase from April for about six months, with the largest number in July (9485 cases) and August (10,386 cases), and the lower incidence occurred in December (2948 cases) (Figure 5). Three peaks of typhoid and paratyphoid were observed in 1999 and 2003. Two were observed every year from 1991 to 1998 and from 2004 to 2006, with the first one generally being from January to March and the second from May to September. Only one peak from July to September was observed in 2000 and 2001 and every year from 2007 (Figure 5).

**Figure 6:**
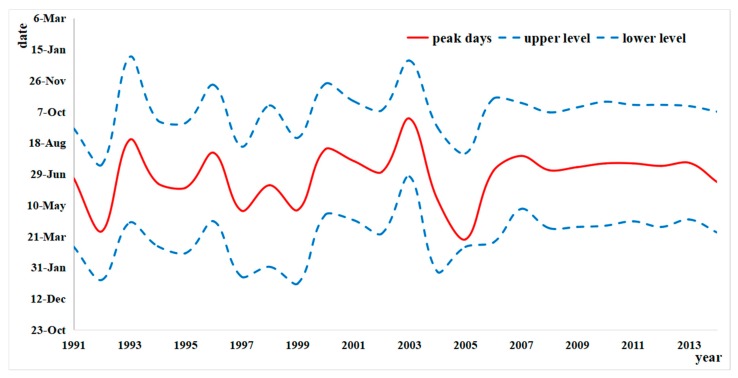
Peak days of typhoid and paratyphoid in Zhejiang from 1991 to 2014 (The peak days, the upper level, and lower level may occur in another year, so the y-axis concludes three years).

As shown in circular distribution, peak days of diseases were not obvious before 2005, as the incidence stayed at a similarly high level through all the months from March to September. However, most peaks occurred in July from 2006, generally appearing on July 16. In the past five years, the peak day of typhoid and paratyphoid was almost July 15 (Figure 6).

#### 3.2.2. Gender Distribution

**Figure 7:**
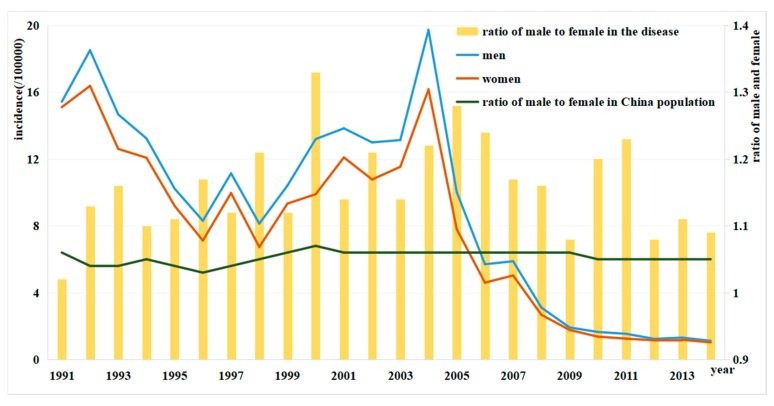
Gender distributions of typhoid and paratyphoid patients in Zhejiang from 1991 to 2014.

The incidences of diseases in males were higher than in females, and the total male-to-female ratio was 1.16:1, with the high ratios occurring in 2000 (1.33:1), 2005 (1.28:1), and 2006 (1.24:1), and the low ratios in 1991 (1.02:1), 2009 (1.08:1), and 2012 (1.08:1). Expect for 1991, the male-to-female ratios for typhoid and paratyphoid were higher than the male-to-female ratios in China population (Figure 7).

#### 3.2.3. Occupational Distribution

**Figure 8:**
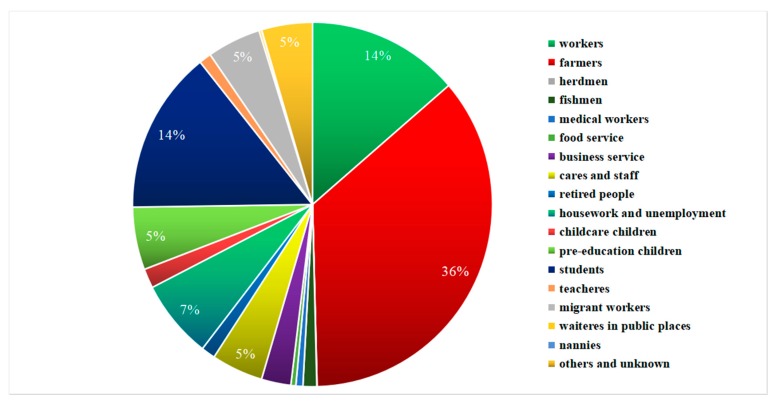
Occupational distribution of typhoid and paratyphoid in Zhejiang from 1991 to 2014.

**Figure 9:**
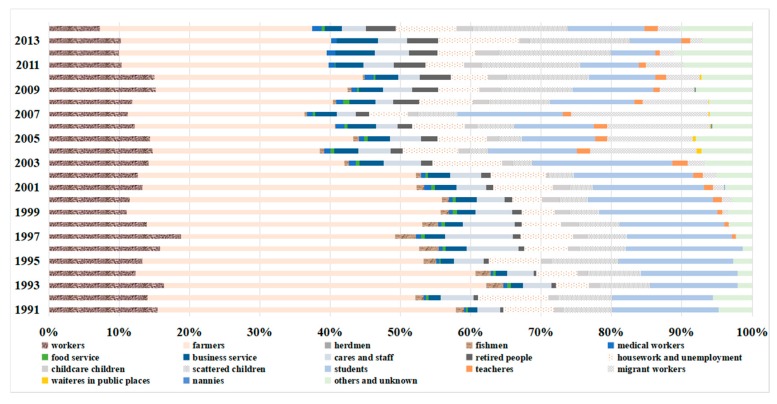
The proportion of different careers in all patients in Zhejiang from 1991 to 2014.

The incidences differed by occupation, with farmers (36%), students (14%), and workers (14%) accounting for more than 50% of all patients (Figure 8). The proportion of farmers gradually declined from 42.44% and remained around 30%, the proportion of workers declined from 15.16% to 10.9%, but pre-education children showed a rising pattern starting in 2004, reaching 13.58% till 2014 (Figure 9).

#### 3.2.4. Age Distribution

**Figure 10:**
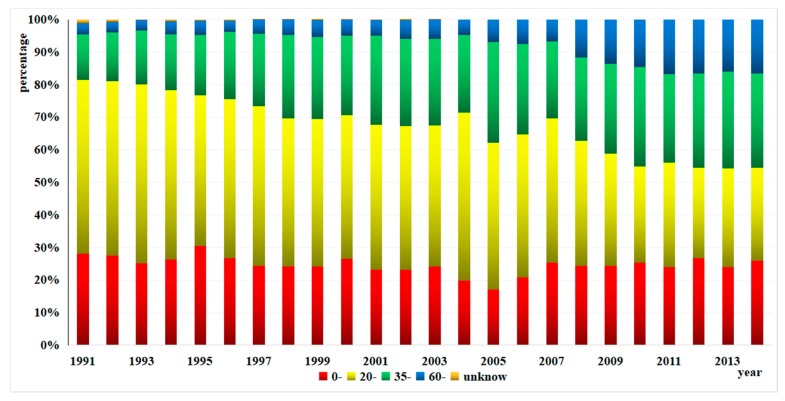
Proportion of the cases in each age group from 1991 to 2014.

The main typhoid and paratyphoid population were aged from 20 to 59 years, but the proportion of those aged over 60 rose gradually from 3.35% to 16.63%, and the proportion of those between 35 and 59 rose from 14.02% to 28.87%, while the proportion of those between 20 and 34 declined by a large margin from 53.28% to 28.49% (Figure 10).

## 4. Discussion

In this study, we investigated the epidemiological trend of typhoid and paratyphoid fevers from 1953 to 2014, and further explored their temporal and spatial distribution, correlation between altitude and incidence, and characteristics regarding gender, occupation, and age.

The incidences of typhoid and paratyphoid fevers have fallen dramatically, and their fatality rate has declined since 1953, as reflected in the findings of a national investigation by Yan et al. [5] and a local one in Deqing County by Xu [17]. Intestinal infectious diseases in China have been significantly reduced since the implementation of the Patriotic Health Campaigns by the National Patriotic Health Committee and the health department in the 1970s and the introduction of the healthy policy of tubing water and toilet [18,19]. The measures taken for tubing water and toilet have resulted in declining incidences of intestinal infectious diseases of various degrees in Jiaxing and Yuhuan [20,21].

The Patriotic Health Campaign launched by the government at the beginning of the foundation of the People’s Republican of China helped reduce the incidence of all kinds of diseases and significantly improved public health [22]. The incidence in 1969 fell to the lowest during the Cultural Revolution, which might be attributed to the fact that all the work in health epidemic prevention stations was stopped and case reports were incomplete. The same was true with Zhejiang Province. The outbreak of chloramphenicol-resistant M1 bacteria in 1988 led to the highest incidence in the history [23]. In 2004, the China Information System for Diseases Control and Prevention was introduced and disease reporting became more convenient and reliable, which led to an abrupt increase in reporting and a false peak. Thanks to all the preventive measures taken for many years, the incidence was found to be 0.95 per 100,000 population in Zhejiang in 2014, indicating the province as a low-level area of typhoid and paratyphoid.

The plain areas are more greatly affected by heavy rains, typhoons, and floods than the mountainous and hilly areas in the summer and autumn, resulting in a higher probability of polluted water in low-altitude areas than in those of high altitude. Further, people in the plain drink river water, while those in the mountainous and hilly areas drink well water [24]. These factors could explain the phenomenon that the incidences of typhoid and paratyphoid were lower in high-altitude areas than in the low-altitude plain in Zhejiang. Consistent with most studies [25,26,27], the high incidence of disease gradually moved to the southeast coastal areas such as Taizhou, Wenzhou, and Ningbo from the north areas in Zhejiang around 1993. Zhoushan, Ningbo, and Taizhou were high-risk areas in the province, which was different from the previous results that Taizhou, Wenzhou, Jinhua, and Ningbo were the areas with high incidence of typhoid and paratyphoid in Zhejiang [28]. The incidence of typhoid and paratyphoid in Ningbo has always been on the top of the list in Zhejiang since 1953, probably because the residents there have the habit of eating pickled raw or half-cooked seafood. The incidence in Zhoushan increased sharply to the highest in 1997 and by a large margin in 1998, possibly because of the continuous drought and lack of water. As an island, the disease prevention and control departments in Zhoushan pay much attention to drinking water disinfection and control the probable outbreak of typhoid and paratyphoid caused by water pollution after typhoons and storms [29]. Similar to many other areas [30,31,32], typhoid and paratyphoid could occur all year in Zhejiang, and the peak incidence often occurs in the summer and fall. The peak period every year was found to be in the months from April to October, which was different from the study by Zhou [33], who reported that the peak period was from July to November when he analyzed the epidemiological characteristics of typhoid and paratyphoid in Xichang Municipality. The main reason for the high incidence in summer and autumn may be the influence of extreme weather such as typhoon in this period. The peak day of incidence in Zhejiang occurred in every month from March to September before 2006, after which the peak period fell around July 15. Some reasons could account for this phenomenon. The peak period was not stable until 2007. In principle, circular distribution is generally applicable to the disease that has only one peak period, which may explain the fluctuation of the peak day before 2007. This might be associated with the establishment of the China Information System for Diseases Control and Prevention, which makes timely case reporting possible so that information can be retrieved and analyzed immediately.

The majority of typhoid and paratyphoid cases were aged from 20 to 59 years. Zhejiang, due to rapid economic development, has a great number of migrant workers, and both aggregation and migration of the population could lead to the high incidence of typhoid and paratyphoid. The proportion of the patients over 60-years old increased yearly probably because of the weakening immunity of these people. Consistent with other intestinal infectious diseases [34,35], the incidences of typhoid and paratyphoid were higher in men than in women. An explanation may be found in the fact that men might be engaged in more outdoor activities, had poorer hygiene habits, and ate more casually than women [36,37]. When it comes to career, farmers, students, and workmen had higher incidences than others, which was close to other studies [32,38,39]. The high percentage found for farmers may due to such factors as drinking water without disinfection or with incomplete disinfection, poor sanitation (outdoor latrines and widespread rubbish heaps), lack of health consciousness, and unhygienic diet.

## 5. Conclusions

The incidence of typhoid and paratyphoid fevers was found to decline and reached a low level in Zhejiang in 2014 after several large fluctuations, with peak periods falling in the months from April to October every year. The findings showed that those aged over 35 years, male, and farmers in particular should be effectively intervened early via measures such as surveillance and health education. The high-risk areas like the southeast coastal areas in Zhejiang Province should be strictly monitored, especially in peak periods, every year. The health authorities should make efforts to protect high-risk populations and further reduce the incidences of these diseases by strengthening measures to improve water and lavatories and helping residents develop good hygiene habits such as washing hands before eating and after using the bathroom.

## Figures and Tables

**Table 1:** Correlation between the annual incidence of typhoid and paratyphoid and altitude in Zhejiang Province from 1953 to 2014.

Year	*r*	*p*
1953–1957	−0.609	0.047
1958–1962	−0.662	0.026
1963–1967	−0.665	0.026
1968–1972	−0.657	0.028
1973–1977	−0.681	0.021
1978–1982	−0.664	0.026
1983–1987	−0.640	0.034
1988–1992	−0.746	0.008
1993–1997	−0.721	0.012
1998–2002	−0.649	0.031
2003–2007	−0.116	0.734
2008–2012	−0.257	0.445
2013–2014	−0.094	0.783

**Table 2:** The average annual incidence of typhoid and paratyphoid in all municipalities in Zhejiang from 1953 to 2014 (/100,000).

Year	Municipality										
	Southeast Coastal Areas				North Areas			Mid-West Areas			
	Ningbo	Wenzhou	Zhoushan	Taizhou	Jiaxing	Huzhou	Shaoxing	Hangzhou	Jinhua	Quzhou	Lishui
1953-	30.96	10.70	7.74	14.72	39.64	-	6.18	10.45	5.15	-	2.25
1958-	15.81	9.25	8.92	11.76	26.38	-	6.95	9.45	4.06	-	2.70
1963-	13.27	4.17	4.01	4.55	13.04	-	6.90	8.36	2.75	-	0.97
1968-	3.01	0.59	2.79	2.86	9.45	-	3.22	5.77	0.94	-	0.07
1973-	4.57	0.79	2.77	1.41	9.20	-	1.86	3.90	0.56	-	0.09
1978-	18.44	2.04	8.12	2.27	9.59	-	3.56	4.76	0.68	-	0.13
1983-	8.98	0.54	7.14	3.59	31.84	29.40	5.07	9.97	2.87	0.85	0.44
1988-	27.30	16.90	28.78	4.94	51.56	50.39	11.35	9.12	1.83	3.74	0.38
1993-	14.92	11.69	63.92	3.35	28.07	23.06	10.81	4.65	1.53	1.91	0.43
1998-	15.95	7.24	29.01	9.81	9.28	9.10	23.54	4.03	14.99	3.37	0.70
2003-	12.04	10.94	7.08	22.68	2.60	3.39	11.42	4.60	8.11	3.94	0.88
2008-	2.57	2.15	1.48	1.56	0.85	1.16	2.00	1.06	1.62	0.95	0.60
2013-	2.47	1.68	0.47	0.61	0.51	0.36	0.73	0.66	0.54	0.73	0.23
